# Turning over New Leaves

**Published:** 1888-04-28

**Authors:** 


### Turning over New Leaves.

[Books for Review should be sent to The Editor, The Lodge, Porchester Square, W.]

### Dr. Holmes in England.*

When one's friends go abroad one naturally likes to hear all their adventures. These may not be very striking, and the traveller need not have wandered in "fresh fields and pastures new;" there is no track so beaten that fresh eyes will not discover on it a flower, or at least a blade of grass, that was not marked before. In scientific works alone do we want facts in all their bare severity, in all other books we are more interested in the way the facts are seen. Zola defines the novel admirably when he calls it "a corner of the world seen through a temperament," and we may with justice extend the definition to all narratives which partake in any degree of a biographical nature. Novel, poem, essay on any subject under the sun, record of travel or adventure—we shall find the personality of the writer peeping out from all; consciously or unconsciously his temperament will affect his work; it will betray itself in what he sees and how he sees it, and, above all, in what he fails to see. Do not say, O tourist, who has been everywhere under the guidance of the benignant Cook, that when you were in Rome, or Paris, or Norway, you saw everything. You could not; no man can, and you saw just what you were able to see—much or little according to the extent of your soul-vision, but never—though you have climbed every mountain, sailed on every river, "done" every cathedral and picture gallery in the world—never, never the whole. People are long-sighted and short-sighted in mind as well as in body, and each must be content to accept the limits of his qualities, to miss what another sees, consoled by the reflection that he sees what another misses. Therefore, if a friend of ours goes no further afield than Margate, we like to hear what he saw there; it will be a little different from what we chanced upon. For most of us, this exchange of gossip, or at most a few letters, forms sufficient publication of our widest travels; but a man who has several thousand friends whom he has never seen cannot stop at this. All these friends want to know where his tour led him, and how he enjoyed it; and it is impossible to write autograph accounts to each and all. Even supposing that flesh and blood could stand the effort, he doesn't know their names and addresses. This was Dr. Holmes' plight when he sat down to write "Our Hundred Days in Europe;" he could not write letters to all his friends—the friends of the autocrat, and the professor, and the poet—so he wrote a book, which the said friends have appreciated to the extent of sending it into a third edition. The hundred days, of which Britain had ninety-three, and France seven, were not the first that Dr. Holmes had spent in the mother-country. More than fifty years ago an American medical student, who was "grinding bones," and indulging in a good deal of trituration of the same stamp in Paris, refreshed himself by a summer tour through England and Scotland, of which a brief *résumé*—the things whose memory has stood the test of half-a-century of busy life—is given in the prefatory chapter of the present work. These are varied if not numerous—the emotions roused by Stratford-on-Avon, the Derby Day, Vauxhall Gardens, the beauty of Edinburgh. He was, he says, invited to just five entertainments in Britain during that visit. Among these was lunch with Mrs. Macadam, who, he says, gave him bread, and not a stone; and breakfast with Dr. Robert Knox, the Edinburgh anatomist, who had seen Waterloo, and who subsequently got mixed up, to his detriment, with the Burke and Hare murders. Does Dr. Holmes know, we wonder, of Knox's last connexion with America? It was after that Burke and Hare business had ruined him, and it consisted in being showman to a troupe of *soi-disant* Ojibbeway Indians! Of a very different character was this visit. Not only is he asked to more entertainments than he can possibly attend; the invitations are so numerous that he has to engage a secretary to decline them for him. Dr. Holmes is a man whom England delights to honour. He is emphatically an American gentleman, and that is one of the finest evolutionary products of the human species. Besides the social claims which this fact gives, the medical world was pleased to welcome the Harvard Professor, and the literary world crowded round the most genial of our social philosophers. Dr. Holmes belongs to all worlds except *le monde où l'on s'ennuie*, and all pressed their claims upon him. Therefore this book becomes more or less of a record of festivities at all manner of houses. The only kind of entertainment he did not appreciate to the full was the garden party. "They are all very well for young people," he says, "and for those who do not mind the nipping and eager air with which the climate of England, no less than that of America, falsifies all the fine things the poets have said about May and even June." Besides all these social functions, Dr. Holmes went to a Derby and saw Ormonde win a race which was not unworthy of his recollections of the one of 1834. In connexion with this, he says, "Every New England deacon ought to see one Derby day to learn what sort of a world this is he lives in. Man is a sporting as well as a praying animal." But as it chances, the praying side of man's nature often says very hard things of the sporting side; and the praying proportion is so much the stronger in the average deacon that it is probable even a New England specimen of the race would be more scandalised than excited at the spectacle; or if he did enjoy it, how horribly ashamed of himself he would be next day! Besides this, we are told of the reception at the three universities, each of which conferred an honorary degree on Dr. Holmes—Oxford, Cambridge, and Edinburgh; of the Cambridge undergraduate who raised a shout of laughter by asking if he came in the famous "one-hoss shay"; and the Edinburgh students who demanded a speech with a persistence which reminded the doctor of the crowd which once for about the space of two hours cried out, "Great is Diana of the Ephesians," and with Doric obstinacy kept shouting till their demand was complied with. As for Oxford, the gown in which he was arrayed there was so gorgeous, that Dr. Holmes felt "as if Solomon in all his glory was not arrayed like the candidates for the degree of D.C.L." In the midst of these various excitements, however, there was time found for a quiet week at Stratford-on-Avon, where the boys showed "a plentiful lack of intelligence" on being asked who Shakespeare was. Another quiet time was spent at Salisbury, and a visit to Stonehenge formed the inspiration of the poem that was read to those "Boys of Twenty-nine," of whom their old mate has so often told us. Eight met to hear it; eight out of the once large joyous gathering who kept up this yearly festival in memory of their college days. At such banquets as these memory becomes more and more the *pièce de résistance* as the years go on. Only eight! Yes, the broken circle at Stonehenge was a fair enough parallel in most things, but not in all. The last stanzas of the poem tell wherein a contrast lies :—

"Cold is the Druid's altar stone,
Its vanished flame no more returns;
But ours no chilling damp has known—
Unchanged, unchanging, still it burns.

"So let our broken circle stand,
A wreck, a remnant, yet the same,
While one last, loving, faithful hand
Still lives to feed its altar flame."

* "Our Hundred Days in Europe." by Oliver Wendell Holmes. London: Sampson Low, Marston, Searle, and Rivington.

Of the many professional brethren whom he met, Dr. Holmes makes special mention of Dr. Priestley, who, with his wife, did much to make his stay in London pleasant, even to giving up his house to Dr. Holmes and his daughter to hold a reception in: housekeepers know how sincere a friendship such a piece of courtesy displays. He also gives Mr. Lawson Tait some words of praise, which should not fail to gratify him. "Mr., or more properly Dr., Tait has had the most extraordinary success in a class of cases long considered beyond the reach of surgery. If I refer to it as a scientific *hari kari*, not for the taking but the saving of life, I shall come near enough to its description. Mr. Lawson Tait has had, so far as I have been able to learn, the most wonderful series of successful cases on record, namely, one hundred and thirty-nine operations without a single death." The other people in whom Mr. Holmes was most interested were his contemporaries, Mr. Gladstone, Lord Tennyson, and others who first drew breath in the year 1809. It is natural that he should take a special interest in these; but his faculty for appreciating all the people he met, the fair ladies and clever men, is youthful in its fresh readiness. Reading the book, one shares in that enjoyment of the London season which he took with a zest as real as if he had been a girl of eighteen, but more appreciative—tasting the different flavours in a fashion unknown to the hungry palate of youth. This is the charm of this narrative of the hundred days—that it makes us feel the writer's enjoyment. It is not written for all time, but for an age—the age, namely, when a large number of people on both sides of the Atlantic regarded as a friend the man who had given them so often a delightful mélange of science, poetry, philosophy. As a gift from that friend we receive it gratefully.

### Human Anatomy and Physiology.*

The teaching of anatomy and physiology separately as two distinct subjects is no doubt quite necessary, and is the course almost universally adopted. But the teaching of the two in combination as one subject has the decided advantage of being more interesting and much more satisfying to the scientific sense of the fitness of things. Professor Witowski has now reached Part XI. of his great work. The method of instruction by superposed plates is not new, but is very effective for certain readers. No man can *learn* anatomy or physiology by such plates alone, any more than he can learn the one or the other by any "book" method whatsoever. It is impossible for anyone to acquire a really practical and working knowledge of such subjects without careful dissection in the case of anatomy, and intelligent experimentalism in the case of physiology. But for those who have passed through the ordinary medical curriculum, and done their work intelligently and industriously, coloured superposed plates such as are here employed are invaluable. As refreshers of the memory, and as correctors and completers of knowledge, the plates, with the accompanying letterpress, may be said to be absolutely essential to those who, desiring to maintain an intelligent familiarity with anatomical and physiological facts, are yet entirely removed by circumstances from the practical work of the laboratory. Both the plates and the letterpress are well done, as is also the translation; and we have no hesitation in recommending the work to those who, not content to be drudges, are anxious to pursue their practice with conscientious intelligence.

* "Human Anatomy and Physiology." Part XI. By Professor G. J. Witowski, M.D. Translated by R. Milne Murray, M.A., M.B., F.R.C.P.E. London: Baillière, Tindal, and Cox.

